# EGFR pathway alterations correlate with rapid early progression in glioblastoma

**DOI:** 10.1007/s11060-025-05282-9

**Published:** 2025-11-10

**Authors:** Kayla Samimi, Aren Singh Saini, Kaylie Cullison, Janette Herr, Eric A. Mellon

**Affiliations:** 1 Henry Ford Health + Michigan State University Health Sciences, Detroit, MI, USA; 2 Department of Radiation Oncology, Henry Ford Cancer Institute, Henry Ford Health, Detroit, MI USA; 3 Department of Radiology, College of Human Medicine, Michigan State University, East Lansing, MI USA; 4 Department of Radiation Oncology, Sylvester Comprehensive Cancer Center, University of Miami Miller School of Medicine, Miami, USA

**Keywords:** Glioblastoma, Rapid early progression, EGFR, Recurrence

## Abstract

**Purpose:**

Rapid early progression (REP) is defined as MRI progression of glioblastoma after surgical resection before adjuvant therapy. REP occurs in approximately 50% of glioblastoma and is associated with worse overall survival. Despite the increasing trend towards molecular characterization of glioblastoma, no analysis has been performed between molecular alterations in glioblastoma and REP that may help define the urgency of adjuvant therapy.

**Methods:**

A retrospective review of 100 consecutive glioblastoma patients who underwent gross or subtotal resection followed by next generation sequencing and radiation therapy (RT). REP was defined as increased nodular enhancement at the resection cavity border between postoperative and RT planning MRI. Next generation sequencing identified alterations in the following genes in at least 10% of patients: TERT promoter, EGFR, PTEN, TP53, CDKN2A/2B, NF1, MTAP, PIK3CA. In addition to MGMT methylation status and patient characteristics, these were tested for correlation with REP and overall survival.

**Results:**

REP was observed in 45 patients. On univariate analysis, EGFR alterations (*p* = 0.001) and MGMT methylation (*p* = 0.016) were significantly associated with REP. On multivariate analysis, only EGFR alterations (*p* = 0.006) remained associated with REP. No correlations were identified with overall survival and REP, EGFR, or MGMT methylation. Of note, there were statistically significant correlations between overall survival and NF1, TERT, MGMT methylation, and degree of resection.

**Conclusion:**

This study identifies EGFR alterations as a significant predictor of REP in glioblastoma. Patients with these alterations identified post-operatively may benefit from expedited adjuvant therapy to mitigate early tumor progression. Further research is needed to refine treatment strategies for this high-risk subset.

## Key points

Rapid early progression occurs in 50% of patients with glioblastoma.

Rapid early progression was associated with presence of EGFR alterations in glioblastoma.

## Importance of study

Rapid early progression (REP) is radiologically defined as tumor regrowth between postoperative imaging and radiotherapy initiation. Although occurring in nearly 50% of glioblastoma patients, it is poorly understood. Molecular biomarkers that drive REP have yet to be analyzed. Our study shows that alterations in EGFR, one of the most common pathways in glioblastoma, may be a driver of REP. This and studies like it may help to inform treatment decisions and warrant acceleration of adjuvant therapy in this subgroup.

## Introduction

Glioblastoma is the most aggressive and common primary brain tumor in adults characterized by rapid growth and poor prognosis. Current standard of care for glioblastoma involves surgical resection followed by RT with both concurrent and adjuvant temozolomide [1]. Despite advances in surgical resection, radiotherapy, and chemotherapy, the median survival remains approximately 16–21 months [2].

Molecular profiling of glioblastoma has been integrated into management to classify patients into molecular subtypes [3, 4]. Common alterations seen in glioblastoma include epidermal growth factor receptor (EGFR), O-methylguanine-DNA methyl-transferase (MGMT) promotor methylation, p53, among others. Understanding these genomic alterations and their implications has been a large area of study in glioblastoma. For instance, EGFR is a tyrosine kinase receptor altered in 40–45% cases of glioblastoma. EGFR dysregulation, particularly amplifications and point deletions, has been implicated in enhancing tumor invasion and resistance to radiation and chemotherapy [5]. Additionally, MGMT methylation, seen in 45% of cases, has been shown to increase overall survival (OS) due to improved response to temozolomide [2, 6].

**Fig. 1:**
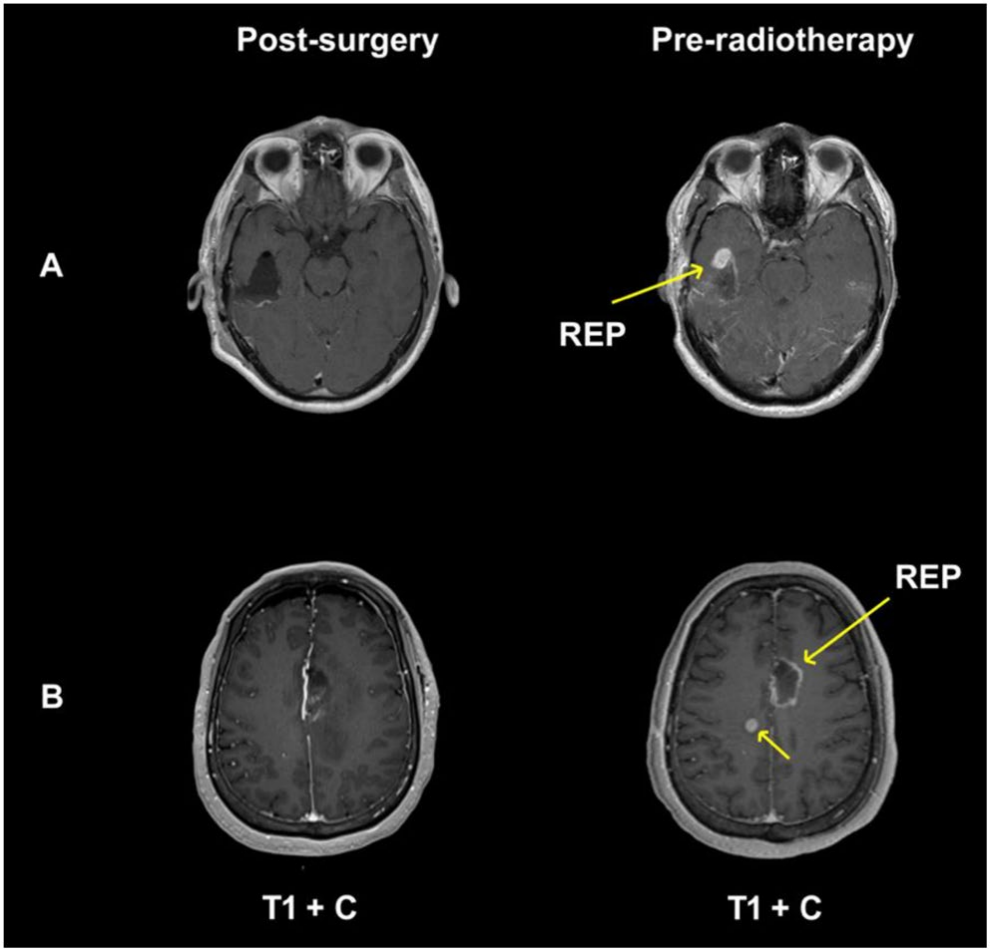
T1 gadolinium enhanced MRIs of two sample patients with newly diagnosed glioblastoma who demonstrate rapid early progression after gross total resection. (**A**) 37-year-old male who underwent right temporal craniotomy (post-surgery top left) demonstrates REP on pre-RT MRI (top right) with nodular lesion extending anteriorly and inferiorly along resection cavity. (**B**) 64-year-old woman who underwent left frontal craniotomy (bottom left) demonstrates REP on pre-RT MRI (bottom right) with recurrence of tumor along the posterior surgical cavity and new right posterior frontal centrum semiovale lesion measuring 1 cm

Glioblastoma’s infiltrative growth and therapeutic resistance are compounded by frequent recurrence, which remains a critical barrier to improving outcomes. Among recurrence patterns, rapid early progression (REP), radiologically defined as tumor regrowth between postoperative imaging and RT initiation, has emerged as a particularly aggressive phenotype. Prior studies have shown that approximately 50% of glioblastoma patients demonstrate REP [7–9]. (Fig. 1) REP has already been shown to be associated with worse prognosis and decreased OS [7–13]. Yet to our knowledge, no studies to date have analyzed the molecular drivers of REP in glioblastoma patients, which might determine the urgency of adjuvant therapies after surgery. The purpose of this study is to therefore understand the association between genetic glioblastoma alterations and presence of REP.

## Methods

A total of 100 patients with a primary diagnosis of glioblastoma, confirmed by pathology, treated at one tertiary center between 2018 and 2023 were included in this study. All patients were diagnosed with IDH wild-type glioblastoma in line with the 2021 WHO Classification Criteria. All patients underwent gross total resection (GTR) or subtotal resection (STR) followed by radiotherapy. Patients that received biopsy without resection were excluded from the study to ensure accurate reporting of REP. Extent of resection was determined radiographically. Additionally, all patients received both post-operative MRI and repeat MRI prior to the start of radiotherapy (pre-RT MRI). Of note, obtaining pre-RT MRI was standard of care at our institution for all patients treated for glioblastoma.

REP was defined as increased nodular enhancement at a distant site or at the resection cavity on pre-RT MRI. This definition was consistent with previously established definitions in literature. MRIs were acquired in thin-cut volumetric fashion and analyzed by overlays in MIM software. The primary imaging sequence used to evaluate for REP was the T1-weighted contrast-enhanced scan. Additional sequences, including T2-weighted, T2 fluid-attenuated inversion recovery (FLAIR), and diffusion-weighted imaging (DWI), were reviewed to aid in interpretation when unclear but were not used as primary criteria. Radiology reports were analyzed to indicate growth of existing enhancement or new enhancement and reviewed by the study team to confirm. Of note, patients diagnosed with REP vs. non-REP both received standard of care of concurrent radiotherapy with temozolomide and adjuvant temozolomide. Therapy was not altered based on presence of REP per institutional protocol.

**Fig. 2:**
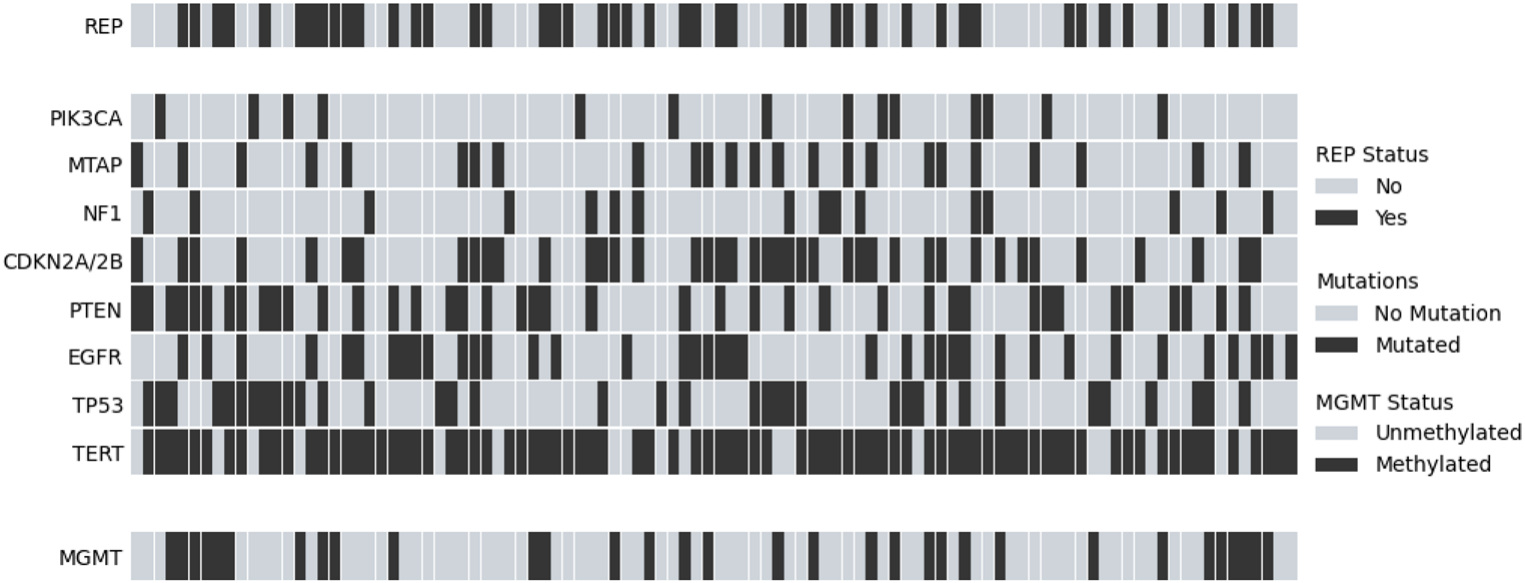
Heatmap showing all 100 glioblastoma patients with REP status and gene alterations (selected for 10% frequency or higher)

Clinical and molecular data was collected from patient records, including demographics (age, sex, race), Karnofsky Performance Scale (KPS) at initiation of RT, and OS. TTFields was not analyzed in this patient population as its use as first-line therapy was < 10%. Next-generation sequencing (NGS) was performed by Foundation Medicine or Caris clinical assays on tumor tissue samples following resection. FoundationOneCDx utilized targeted assays and Caris MI Tumor Seek Hybrid utilized whole exome sequencing to generate reports [14, 15]. Using the distribution of REP and non-REP in this dataset, a pathway alteration that perfectly correlates with REP or non-REP would have to be present in at least 9% of the population to be statistically significant on univariate analysis. Therefore, genes altered with a prevalence of at least 10% were included in analysis: EGFR, TERT, PTEN, TP53, CDKN2A/2B, NF1, MTAP, and PIK3CA. MGMT promoter methylation status was determined using methylation-specific PCR (Fig. 2). All alterations were known or suspected pathogenic alterations per vendor provided annotated reports.

For statistical analysis, SPSS (version 29.0.2.0) was used to perform chi-square tests and survival analyses to evaluate relationships between OS, REP, and pathogenic or possibly pathogenic NGS findings. OS was defined as time from surgical resection until date of death or censored at last follow-up. Chi-square analysis was used for categorical variables and Mann-Whitney U test was used for continuous variables. Kaplan-Meier method was used to plot survival curves with log-rank test to determine the significance of differences in survival distributions. The Cox proportional hazard model was used to perform univariable and multivariable regression. A p-value < 0.05 (two-sided) was considered statistically significant for all analysis.

## Results

Of the 100 included patients, 45 met the criteria for REP. REP/non-REP groups were comparable with respect to age (*p* =0.45), gender (*p* =1), race (*p* = 0.27) extent of surgical resection (*p* = 1), and KPS score (*p* = 0.869) (Table 1). Median survival time for all patients was 18.7 months and median age was 61 years. The median time from postoperative MRI to pre-RT MRI was similar between groups (REP: 27 days (14–76) vs. non-REP: 28 days (16–81); *p* = 0.479), as was time from surgery to radiation initiation (REP: 38 days [17–97] vs. non-REP: 36 days [14–75]; *p* = 0.199).

**Table 1:** Characteristics and demographics of 100 total patients included in study

	REP (*n* = 45)	Non-REP (*n* = 55)	*p*-value
Age (years)			*p* = 0.454
<60	22	25	
>60	23	30	
Sex			*p* = 1.000
Male	26	31	
Female	19	24	
Race			*p* = 0.269
White non-Hispanic	17	27	
White Hispanic	24	26	
Black non-Hispanic	4	1	
Asian	0	1	
Extent of resection			*p* = 1.000
Gross total resection	30	36	
Subtotal resection	15	19	
KPS at the start of RT			*p* = 0.869
≥ 90%	30	36	
≤ 80%	15	19	

No statistically significant differences were noted between REP and non-REP groups when comparing gender, age, race, extent of surgical resection, and KPS score at the start of RT

On chi-square univariate analysis, the presence of EGFR alterations (*p* = 0.001) and MGMT methylation (*p* = 0.016) significantly correlated with REP. After multivariate regression, only EGFR alterations significantly correlated with REP (*p* = 0.009). There was no significant association between REP and alterations in TERT promoter, PTEN, TP53, CDKN2A/2B, NF1, MTAP, and PIK3CA (Table 2). To further assess the effect of the patient characteristics in Table 1, a second multivariate analysis was performed including age, gender, race, surgical extent, and KPS at the start of RT. EGFR alterations remained the only independent predictor of REP (*p* = 0.006), with no significance for any of the other patient characteristics or genetic alterations in Table 1 or 2. EGFR alterations in the cohort included amplification (*n* = 17, 45%), EGFRvIII (*n* = 10, 26%), missense mutations (*n* = 10, 26%), and fusion (*n* = 1, 3%). When analyzed in univariate fashion by mutation type, there was a statistically significant association between REP and EGFR amplification (*p* = 0.031), but not for EGFRvIII (*p* = 0.750) or missense mutations (*p* = 0.108).

**Table 2:** Univariate and multivariate linear regression for all genetic alterations with REP

	REP (*N* = 45)	Non-REP (*N* = 55)	Univariate linear regression	Multivariate linear regression
TERT (*N* = 82)	38 (46%)	44 (54%)	*p* = 0.565	*p* = 0.838
TP53 (*N* = 36)	15 (42%)	21 (58%)	*p* = 0.615	*p* = 0.916
EGFR (*N* = 38)	25 (67%)	13 (33%)	*p* = 0.001**	*p* = 0.009*
PTEN (*N* = 40)	15 (38%)	25 (62%)	*p* = 0.218	*p* = 0.113
CDKN2A/2B (*N* = 41)	21 (51%)	20 (49%)	*p* = 0.297	*p* = 0.190
MGMT (*N* = 32)	20 (63%)	12 (37%)	*p* = 0.016*	*p* = 0.056
MTAP (*N* = 24)	11 (46%)	13 (54%)	*p* = 0.925	*p* = 0.174
NF1 (*N* = 16)	6 (38%)	10 (62%)	*p* = 0.511	*p* = 0.894
PIK3CA (*N* = 14)	4 (29%)	12 (71%)	*p* = 0.183	*p* = 0.595

*p < 0.005

**p < 0.001

There was no statistically significant difference in OS when comparing presence of REP (REP: 22 months vs. non-REP: 23.7 months; *p* = 0.977). There was also no statistically significant difference in OS when comparing presence of EGFR alterations (EGFR alteration: 22 months vs. no EGFR alteration: 22.8 months; *p* = 0.575). There was also no statistically significant difference between OS and EGFR amplification (*p* = 0.889), EGFRvIII (*p* = 0.835), or EGFR missense mutations (*p* = 0.244).

Of note, extent of surgical resection was associated with a significant difference in OS on both univariate (*p* = 0.002) and multivariate cox regression analysis (*p* = 0.003). Median survival in patients with GTR was 25.9 months vs. 17.5 months with STR.

There was a significant relationship between OS and NF1 mutations on both univariate (*p* = 0.004) and multivariate (*p* = 0.004) cox regression analysis. Median survival in patients with mutations in NF1 was 11.7 months, compared to a median survival of 24.5 months in those without the NF1 mutation.

**Fig. 3:**
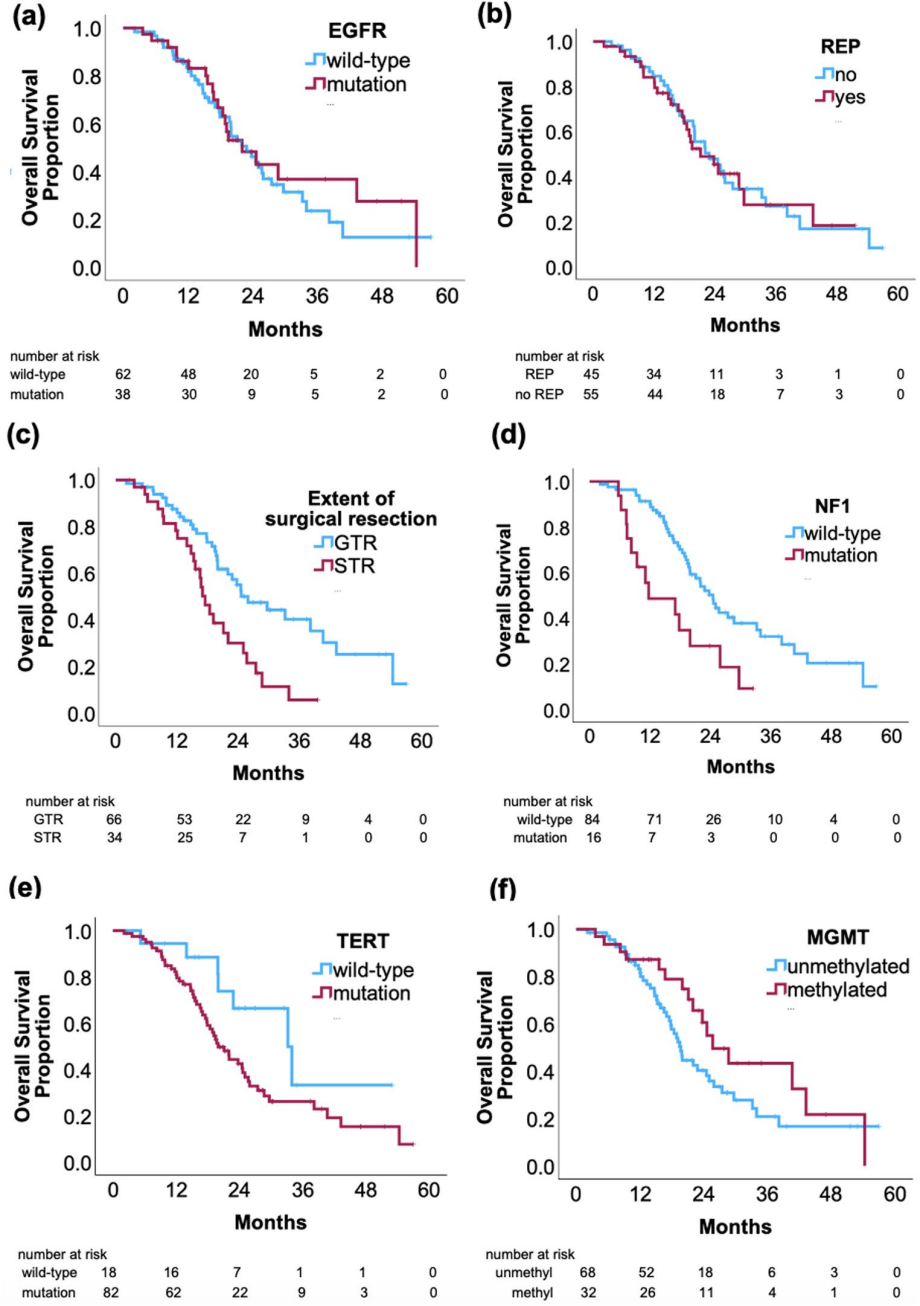
Kaplan Meier survival curves comparing OS with REP, extent of surgical resection, EGFR, NF1, and TERT promoter alterations. There was no significant correlation between EGFR alterations and OS (*p* = 0.575) (**a**) and between OS and REP (*p* = 0.977) (**b**). Significant differences in OS was seen in patients when analyzing extent of surgical resection (*p* = 0.002) (**c**), NF1 mutations (*p* = 0.004) (**d**), TERT promoter mutations (*p* = 0.022) (**e**), and MGMT methylation status (*p* = 0.015) (**f**)

In addition, TERT promoter mutations were associated with a significant difference in OS on multivariate analysis (*p* = 0.022) yet not on univariate analysis (*p* = 0.066). Median survival in patients with TERT promoter mutation was 20.0 months vs. 33.2 months for those without TERT promoter mutation. MGMT methylation was also associated with a significant difference in OS on multivariate analysis (*p* = 0.015) and not on univariate analysis (*p* = 0.106) (Fig. 3). Median survival in patients with MGMT methylation was 31.6 months vs. 25.5 months for those without MGMT methylation. There was no statistically significant association with OS and age, sex, race, or alterations in PTEN, TP53, CDKN2A/2B, MTAP, and PIK3CA (Table 3).

**Table 3:** Univariate and multivariate cox regression analysis for OS. Included in the model is REP, patient characteristics, and alterations in TERT promoter, EGFR, PTEN, TP53, CDKN2A/2B, NF1, MTAP, PIK3CA, and MGMT methylation status

	Univariate Cox regression			Multivariate cox regression		
	Hazard ratio	CI (95%)	p-value	Hazard ratio	CI (95%)	p-value
Age	1.231	0.732–1.072	0.434	1.321	0.713–2.449	0.377
Sex	1.336	0.797–2.239	0.272	1.365	0.689–2.706	0.373
Race	1.155	0.970–1.375	0.105	1.195	0.973–1.469	0.089
GTR vs. STR	2.290	1.343–3.905	0.002*	2.522	1.363–4.667	0.003*
KPS score	0.995	0.964–1.028	0.762	1.008	0.969–1.049	0.695
REP	0.992	0.587–1.676	0.977	1.252	0.672–2.331	0.479
TERT	2.101	0.951–4.638	0.066	2.908	1.143–7.394	0.025
TP53	0.921	0.536–1.585	0.767	1.834	0.907–3.707	0.091
EGFR	0.856	0.497–1.474	0.575	0.816	0.380–1.754	0.603
PTEN	1.119	0.663–1.887	0.674	1.301	0.732–2.313	0.369
CDKN2A/2B	0.762	0.449–1.294	0.315	1.084	0.473–2.485	0.848
MGMT	0.624	0.350–1.111	0.109	0.402	0.194–0.835	0.015*
MTAP	0.838	0.458–1.531	0.565	1.947	0.749–5.063	0.172
NF1	2.465	1.316–4.617	0.005*	3.583	1.553–8.268	0.003*
PIK3CA	1.169	0.603–2.266	0.643	1.105	0.494–2.471	0.808

*p < 0.005

## Discussion

Since REP is observed in nearly 50% of glioblastoma patients, understanding its molecular underpinnings may help guide adjuvant therapy. Our study identifies EGFR alterations, specifically EGFR amplification, as a significant predictor of REP in glioblastoma. The association between EGFR alterations and REP in our cohort aligns with the established role of EGFR in glioblastoma aggressiveness and therapeutic resistance [5].

Characteristics of our study sample are consistent with previously reported cohorts of glioblastoma. Similar to the approximately 50% seen in reported literature, 45% of patients demonstrated REP [7, 8]. Molecular factors were also consistent, including the prevalence of TERT promoter mutations (82%) and EGFR alterations (38%) and the prevalence of mutations such as NF1 (16%) [16, 17].

Clinically, our findings suggest that glioblastoma patients with EGFR alterations may benefit from expedited initiation of adjuvant therapies after surgery to mitigate the risk of rapid tumor progression. Standard treatment typically allows radiotherapy up to six weeks after resection, based on the belief that early radiation may be less effective due to post-surgical hypoxia caused by disrupted vasculature, an environment associated with radiation resistance [18–21]. However, several studies have reported that delays in initiating radiation beyond this window may worsen outcomes and increase mortality [22–24]. This phenomenon may be age-dependent. Bogdan et al. showed decreased OS for patients > 40 years old who began radiation more than 37 days postoperatively [25].

Thus, while the optimal timing of radiation remains debated, our results suggest that in patients with REP, delays may allow for clinically significant progression. Early therapeutic intervention such as radiation dose escalation, a short hypofractionated dosage, targeted therapies, or intensified chemotherapy regimens could be explored. Understanding the molecular drivers of REP may therefore guide not only risk stratification but also personalized timing of adjuvant therapy.

Additionally, obtaining a pre-RT MRI in glioblastoma patients was standard of care at our institution, yet practice varies between institutions. The pre-RT MRI has been advocated for in the literature [13, 26]. Obtaining a pre-RT MRI can help to differentiate REP from progression after starting radiation therapy, which can differentiate REP from treatment resistance [11]. Studies have found glioblastoma doubling time to be 21.1–29.8 days or 1.4–2.1% growth per day, and hence have advocated for routine use of pre-RT MRI to not miss rapidly growing recurrence in RT planning [26].

Furthermore, the treatment of glioblastoma is heavily challenged by the heterogeneous makeup of the tumor [27]. Molecular profiling such as next generation sequencing may therefore underestimate tumor heterogeneity, which limits our analysis. However, studies in other cancers known to be impacted by EGFR alterations, particularly colon cancers and non-small cell lung carcinoma of the lung, have shown clinical indication for EGFR targeted therapy even in cases where all tumor cells do not express the mutation [28]. Therefore, understanding if EGFR is a driver of REP may be helpful to guide therapies and predict patients at high risk for REP in glioblastoma.

Interestingly, while prior studies have demonstrated an association between REP and reduced OS [7], our study did not confirm this relationship. While survival was numerically longer in patients without REP (23.7 months) when compared to patients with REP (22.0 months), this was not statistically significant. This discrepancy may be attributed to the smaller sample size (*n* = 100), single institution nature of this study, or possible patient bias. Nonetheless, the strong correlation between EGFR alterations and REP highlights the potential utility of this biomarker in identifying patients at high risk for early disease progression.

The univariate association between MGMT methylation and REP may appear counterintuitive since MGMT methylation is typically linked to better response to temozolomide and improved OS [6]. However, this analysis is conducted in the immediate post-operative period before start of temozolomide. Because it was not significant on multivariate analysis, the direct relationship of MGMT with REP is unclear.

Of note, mutations in NF1 were incidentally associated with decreased OS in our analysis. NF1 mutations are generally seen in 13% of glioblastoma patients, notably in the mesenchymal subtype of primary glioblastoma [29]. Mesenchymal glioblastoma is an aggressive phenotype with poor prognosis, which may explain significant association with survival [30]. Additionally, the relationship to decreased OS has been demonstrated before between NF1 mutations and both low grade gliomas and glioblastoma [30, 31]. Yet, larger scale studies have shown no survival differences based on NF1 mutation status in glioblastoma [32, 33]. Therefore, our association is not consistent with these larger data sets.

There was also a significant decrease in OS in patients with TERT promoter mutations. TERT promoter mutations are one of the most common mutations in glioblastoma, identified in 70–80% of cases and are associated with poor prognosis [16, 17, 34]. Some molecular studies have shown associations with poor OS in patients with TERT promoter mutations [17, 35]. However, larger scale studies published by the RANO resect team show that absence of TERT promoter mutation does not confer a survival advantage in glioblastoma [34]. Therefore, the small sample size of this study may have served as a confounding factor to the perceived difference in OS. MGMT methylation was additionally associated with improved OS on multivariate analysis. This is consistent with prior literature as MGMT methylation leads to improved response to temoide and can improve OS [lozam^2^, ^6^].

GTR was also associated with improved OS when compared to STR. In general, maximal safe resection is standard of care for glioblastoma and prior studies have shown that GTR in IDH wild-type glioblastoma can lead to improved OS [36, 37]. Hence, our findings are in accordance with existing evidence.

There are several limitations to this study. REP was determined from T1 and T2 MRI imaging which was interpreted by the investigator, radiologists, and radiation oncologists. More reliable results for REP may be seen with the addition of diffusion-weighted MRI and MR-spectroscopy, which were not analyzed in this study [12, 38]. Finally, subgroup analysis of EGFR alterations identified that only amplifications correlated with REP. The small sample sizes for other EGFR alteration groups preclude firm conclusions about their correlations with REP. Additionally, other subgroup analyses may have been under powered due to small sample sizes, increasing potential of type II error. Larger sample sizes could both confirm our findings and allow for further conclusions regarding specific EGFR alterations in early glioblastoma progression.

## Conclusion

Our study highlights the importance of EGFR alterations as potential biomarkers for predicting REP in glioblastoma, which could inform urgency of adjuvant therapy after surgical resection of glioblastoma.

## Data Availability

Data will be made available upon reasonable request.

## References

[1] Bell EH, Pugh SL, McElroy JP, Gilbert MR, Mehta M, Klimowicz AC, Magliocco A, Bredel M, Robe P, Grosu AL, Stupp R, Curran W Jr., Becker AP, Salavaggione AL, Barnholtz-Sloan JS, Aldape K, Blumenthal DT, Brown PD, Glass J, Souhami L, Lee RJ, Brachman D, Flickinger J, Won M, Chakravarti A (2017) Molecular-Based recursive partitioning analysis model for glioblastoma in the Temozolomide era: A correlative analysis based on NRG oncology RTOG 0525. JAMA Oncol 3:784–792. 10.1001/jamaoncol.2016.6020 28097324 10.1001/jamaoncol.2016.6020 PMC5464982

[2] Stupp R, Mason WP, van den Bent MJ, Weller M, Fisher B, Taphoorn MJ, Belanger K, Brandes AA, Marosi C, Bogdahn U, Curschmann J, Janzer RC, Ludwin SK, Gorlia T, Allgeier A, Lacombe D, Cairncross JG, Eisenhauer E, Mirimanoff RO, European Organisation for R, Treatment of Cancer Brain T, Radiotherapy G, National Cancer Institute of Canada Clinical Trials G (2005) Radiotherapy plus concomitant and adjuvant temozolomide for glioblastoma. N Engl J Med 352:987–996 10.1056/NEJMoa043330 10.1056/NEJMoa043330 15758009

[3] Guo J, Fathi Kazerooni A, Toorens E, Akbari H, Yu F, Sako C, Mamourian E, Shinohara RT, Koumenis C, Bagley SJ, Morrissette JJD, Binder ZA, Brem S, Mohan S, Lustig RA, O’Rourke DM, Ganguly T, Bakas S, Nasrallah MP, Davatzikos C (2024) Integrating imaging and genomic data for the discovery of distinct glioblastoma subtypes: a joint learning approach. Sci Rep 14:4922. 10.1038/s41598-024-55072-y 38418494 10.1038/s41598-024-55072-y PMC10902376

[4] Capper D, Jones DTW, Sill M, Hovestadt V, Schrimpf D, Sturm D, Koelsche C, Sahm F, Chavez L, Reuss DE, Kratz A, Wefers AK, Huang K, Pajtler KW, Schweizer L, Stichel D, Olar A, Engel NW, Lindenberg K, Harter PN, Braczynski AK, Plate KH, Dohmen H, Garvalov BK, Coras R, Holsken A, Hewer E, Bewerunge-Hudler M, Schick M, Fischer R, Beschorner R, Schittenhelm J, Staszewski O, Wani K, Varlet P, Pages M, Temming P, Lohmann D, Selt F, Witt H, Milde T, Witt O, Aronica E, Giangaspero F, Rushing E, Scheurlen W, Geisenberger C, Rodriguez FJ, Becker A, Preusser M, Haberler C, Bjerkvig R, Cryan J, Farrell M, Deckert M, Hench J, Frank S, Serrano J, Kannan K, Tsirigos A, Bruck W, Hofer S, Brehmer S, Seiz-Rosenhagen M, Hanggi D, Hans V, Rozsnoki S, Hansford JR, Kohlhof P, Kristensen BW, Lechner M, Lopes B, Mawrin C, Ketter R, Kulozik A, Khatib Z, Heppner F, Koch A, Jouvet A, Keohane C, Muhleisen H, Mueller W, Pohl U, Prinz M, Benner A, Zapatka M, Gottardo NG, Driever PH, Kramm CM, Muller HL, Rutkowski S, von Hoff K, Fruhwald MC, Gnekow A, Fleischhack G, Tippelt S, Calaminus G, Monoranu CM, Perry A, Jones C, Jacques TS, Radlwimmer B, Gessi M, Pietsch T, Schramm J, Schackert G, Westphal M, Reifenberger G, Wesseling P, Weller M, Collins VP, Blumcke I, Bendszus M, Debus J, Huang A, Jabado N, Northcott PA, Paulus W, Gajjar A, Robinson GW, Taylor MD, Jaunmuktane Z, Ryzhova M, Platten M, Unterberg A, Wick W, Karajannis MA, Mittelbronn M, Acker T, Hartmann C, Aldape K, Schuller U, Buslei R, Lichter P, Kool M, Herold-Mende C, Ellison DW, Hasselblatt M, Snuderl M, Brandner S, Korshunov A, von Deimling A, Pfister SM (2018) DNA methylation-based classification of central nervous system tumours. Nature 555:469–474 10.1038/nature26000 10.1038/nature26000 PMC6093218 29539639

[5] Shinojima N, Tada K, Shiraishi S, Kamiryo T, Kochi M, Nakamura H, Makino K, Saya H, Hirano H, Kuratsu J, Oka K, Ishimaru Y, Ushio Y (2003) Prognostic value of epidermal growth factor receptor in patients with glioblastoma multiforme. Cancer Res 63:6962–6970 14583498

[6] Szylberg M, Sokal P, Sledzinska P, Bebyn M, Krajewski S, Szylberg L, Szylberg A, Szylberg T, Krystkiewicz K, Birski M, Harat M, Wlodarski R, Furtak J (2022) MGMT promoter methylation as a prognostic factor in primary glioblastoma: a single-institution observational study. Biomedicines 10. 10.3390/biomedicines10082030 10.3390/biomedicines10082030 PMC9405779 36009577

[7] Palmer JD, Bhamidipati D, Shukla G, Sharma D, Glass J, Kim L, Evans JJ, Judy K, Farrell C, Andrews DW, Wang ZW, Peiper SC, Werner-Wasik M, Shi W (2019) Rapid early tumor progression is prognostic in glioblastoma patients. Am J Clin Oncol 42:481–486. 10.1097/COC.0000000000000537 30973372 10.1097/COC.0000000000000537

[8] Merkel A, Soeldner D, Wendl C, Urkan D, Kuramatsu JB, Seliger C, Proescholdt M, Eyupoglu IY, Hau P, Uhl M (2017) Early postoperative tumor progression predicts clinical outcome in glioblastoma-implication for clinical trials. J Neurooncol 132:249–254. 10.1007/s11060-016-2362-z 28101701 10.1007/s11060-016-2362-z PMC5378726

[9] Lakomy R, Kazda T, Selingerova I, Poprach A, Pospisil P, Belanova R, Fadrus P, Smrcka M, Vybihal V, Jancalek R, Kiss I, Muckova K, Hendrych M, Knight A, Sana J, Slampa P, Slaby O (2020) Pre-Radiotherapy progression after surgery of newly diagnosed glioblastoma: corroboration of new prognostic variable. Diagnostics (Basel) 10. 10.3390/diagnostics10090676 10.3390/diagnostics10090676 PMC7555958 32899528

[10] Farzana W, Basree MM, Diawara N, Shboul ZA, Dubey S, Lockhart MM, Hamza M, Palmer JD, Iftekharuddin KM (2023) Prediction of rapid early progression and survival risk with pre-radiation MRI in WHO grade 4 glioma patients. Cancers (Basel) 15. 10.3390/cancers15184636 10.3390/cancers15184636 PMC10526762 37760604

[11] Kraus RD, Weil CR, Frances Su FC, Cannon DM, Burt LM, Mendez JS (2022) Incidence and extent of disease progression on MRI between surgery and initiation of radiotherapy in glioblastoma patients. Neurooncol Pract 9:380–389. 10.1093/nop/npac044 36134015 10.1093/nop/npac044 PMC9476988

[12] Wee CW, Kim E, Kim TM, Park CK, Kim JW, Choi SH, Yoo RE, Lee ST, Kim IH (2017) Impact of interim progression during the surgery-to-radiotherapy interval and its predictors in glioblastoma treated with temozolomide-based radiochemotherapy. J Neurooncol 134:169–175. 10.1007/s11060-017-2505-x 28547592 10.1007/s11060-017-2505-x

[13] Sackett JJ (2024) Rapid early progression after surgery for IDH-wildtype glioblastoma and the importance of the pre-radiation planning mrI. Int J Radiation Oncol 120:e268-e269

[14] CarisLifeSciences (2025) Caris Molecular Intelligence^®^ Tumor Profiling. https://www.carislifesciences.com/oncology

[15] Foundation Medicine (2025) FoundationOne^®^ CDx technical information

[16] Liu X, Wu G, Shan Y, Hartmann C, von Deimling A, Xing M (2013) Highly prevalent TERT promoter mutations in bladder cancer and glioblastoma. Cell Cycle 12:1637–1638. 10.4161/cc.24662 23603989 10.4161/cc.24662 PMC3680543

[17] Killela PJ, Pirozzi CJ, Healy P, Reitman ZJ, Lipp E, Rasheed BA, Yang R, Diplas BH, Wang Z, Greer PK, Zhu H, Wang CY, Carpenter AB, Friedman H, Friedman AH, Keir ST, He J, He Y, McLendon RE, Herndon JE 2nd, Yan H, Bigner DD (2014) Mutations in IDH1, IDH2, and in the TERT promoter define clinically distinct subgroups of adult malignant gliomas. Oncotarget 5:1515–1525. 10.18632/oncotarget.1765 10.18632/oncotarget.1765 PMC4039228 24722048

[18] Warren KT, Liu L, Liu Y, Strawderman MS, Hussain AH, Ma HM, Milano MT, Mohile NA, Walter KA (2020) Time to treatment initiation and outcomes in high-grade glioma patients in rehabilitation: a retrospective cohort study. CNS Oncol 9:CNS64. 10.2217/cns-2020-0018 33112686 10.2217/cns-2020-0018 PMC7737197

[19] Wehming FM, Wiese B, Nakamura M, Bremer M, Karstens JH, Meyer A (2012) Malignant glioma grade 3 and 4: how relevant is timing of radiotherapy? Clin Neurol Neurosurg 114:617–621. 10.1016/j.clineuro.2011.12.024 22244251 10.1016/j.clineuro.2011.12.024

[20] Luo W, Wang Y (2019) Hypoxia mediates tumor malignancy and therapy resistance. Adv Exp Med Biol 1136:1–18. 10.1007/978-3-030-12734-3_1 31201713 10.1007/978-3-030-12734-3_1

[21] Chedeville AL, Madureira PA (2021) The role of hypoxia in glioblastoma radiotherapy resistance. Cancers (Basel) 13. 10.3390/cancers13030542 10.3390/cancers13030542 PMC7867045 33535436

[22] Do V, Gebski V, Barton MB (2000) The effect of waiting for radiotherapy for grade III/IV gliomas. Radiother Oncol 57:131–136. 10.1016/s0167-8140(00)00257-7 11054516 10.1016/s0167-8140(00)00257-7

[23] Irwin C, Hunn M, Purdie G, Hamilton D (2007) Delay in radiotherapy shortens survival in patients with high grade glioma. J Neurooncol 85:339–343. 10.1007/s11060-007-9426-z 17579810 10.1007/s11060-007-9426-z

[24] De Barros A, Attal J, Roques M, Nicolau J, Sol JC, Cohen-Jonathan-Moyal E, Roux FE (2019) Impact on survival of early tumor growth between surgery and radiotherapy in patients with de Novo glioblastoma. J Neurooncol 142:489–497. 10.1007/s11060-019-03120-3 30783874 10.1007/s11060-019-03120-3

[25] Glinski B, Urbanski J, Hetnal M, Malecki K, Jarosz M, Mucha-Malecka A, Chrostowska A, Jakubowicz E, Fraczek-Blachut B, Dymek P (2012) Prognostic value of the interval from surgery to initiation of radiation therapy in correlation with some histo-clinical parameters in patients with malignant supratentorial gliomas. Contemp Oncol (Pozn) 16:34–37. 10.5114/wo.2012.27334 23788852 10.5114/wo.2012.27334 PMC3687387

[26] Stensjoen AL, Solheim O, Kvistad KA, Haberg AK, Salvesen O, Berntsen EM (2015) Growth dynamics of untreated glioblastomas in vivo. Neuro Oncol 17:1402–1411. 10.1093/neuonc/nov029 25758748 10.1093/neuonc/nov029 PMC4578579

[27] Patel AP, Tirosh I, Trombetta JJ, Shalek AK, Gillespie SM, Wakimoto H, Cahill DP, Nahed BV, Curry WT, Martuza RL, Louis DN, Rozenblatt-Rosen O, Suva ML, Regev A, Bernstein BE (2014) Single-cell RNA-seq highlights intratumoral heterogeneity in primary glioblastoma. Science 344:1396–1401. 10.1126/science.1254257 24925914 10.1126/science.1254257 PMC4123637

[28] Fruh M, Pless M (2012) EGFR IHC score for selection of cetuximab treatment: ready for clinical practice? Transl Lung Cancer Res 1:145–146. 10.3978/j.issn.2218-6751.2012.03.01 25806170 10.3978/j.issn.2218-6751.2012.03.01 PMC4367568

[29] Cerami E, Gao J, Dogrusoz U, Gross BE, Sumer SO, Aksoy BA, Jacobsen A, Byrne CJ, Heuer ML, Larsson E, Antipin Y, Reva B, Goldberg AP, Sander C, Schultz N (2012) The cBio cancer genomics portal: an open platform for exploring multidimensional cancer genomics data. Cancer Discov 2:401–404. 10.1158/2159-8290.CD-12-0095 22588877 10.1158/2159-8290.CD-12-0095 PMC3956037

[30] Scheer M, Leisz S, Sorge E, Storozhuk O, Prell J, Ho I, Harder A (2021) Neurofibromatosis type 1 gene alterations define specific features of a subset of glioblastomas. Int J Mol Sci 23. 10.3390/ijms23010352 10.3390/ijms23010352 PMC8745708 35008787

[31] Vizcaino MA, Shah S, Eberhart CG, Rodriguez FJ (2015) Clinicopathologic implications of NF1 gene alterations in diffuse gliomas. Hum Pathol 46:1323–1330. 10.1016/j.humpath.2015.05.014 26190195 10.1016/j.humpath.2015.05.014 PMC4703095

[32] Jonsson P, Lin AL, Young RJ, DiStefano NM, Hyman DM, Li BT, Berger MF, Zehir A, Ladanyi M, Solit DB, Arnold AG, Stadler ZK, Mandelker D, Goldberg ME, Chmielecki J, Pourmaleki M, Ogilvie SQ, Chavan SS, McKeown AT, Manne M, Hyde A, Beal K, Yang TJ, Nolan CP, Pentsova E, Omuro A, Gavrilovic IT, Kaley TJ, Diamond EL, Stone JB, Grommes C, Boire A, Daras M, Piotrowski AF, Miller AM, Gutin PH, Chan TA, Tabar VS, Brennan CW, Rosenblum M, DeAngelis LM, Mellinghoff IK, Taylor BS (2019) Genomic correlates of disease progression and treatment response in prospectively characterized gliomas. Clin Cancer Res 25:5537–5547. 10.1158/1078-0432.CCR-19-0032 31263031 10.1158/1078-0432.CCR-19-0032 PMC6753053

[33] Dono A, Ramesh AV, Wang E, Shah M, Tandon N, Ballester LY, Esquenazi Y (2021) The role of RB1 alteration and 4q12 amplification in IDH-WT glioblastoma. Neurooncol Adv 3:vdab050. 10.1093/noajnl/vdab050 34131647 10.1093/noajnl/vdab050 PMC8193911

[34] Karschnia P, Young JS, Dono A, Hani L, Juenger ST, Sciortino T, Bruno F, Teske N, Morshed RA, Haddad AF, Zhang Y, Stoecklein S, Vogelbaum MA, Beck J, Tandon N, Hervey-Jumper S, Molinaro AM, Ruda R, Bello L, Schnell O, Esquenazi Y, Ruge MI, Grau SJ, van den Bent M, Weller M, Berger MS, Chang SM, Tonn JC (2022) TERT promotor status does not add prognostic information in IDH-wildtype glioblastomas fulfilling other diagnostic WHO criteria: A report of the RANO resect group. Neurooncol Adv 4:vdac158. 10.1093/noajnl/vdac158 36325373 10.1093/noajnl/vdac158 PMC9616057

[35] Labussiere M, Di Stefano AL, Gleize V, Boisselier B, Giry M, Mangesius S, Bruno A, Paterra R, Marie Y, Rahimian A, Finocchiaro G, Houlston RS, Hoang-Xuan K, Idbaih A, Delattre JY, Mokhtari K, Sanson M (2014) TERT promoter mutations in gliomas, genetic associations and clinico-pathological correlations. Br J Cancer 111:2024–2032. 10.1038/bjc.2014.538 25314060 10.1038/bjc.2014.538 PMC4229642

[36] Gessler F, Bernstock JD, Braczynski A, Lescher S, Baumgarten P, Harter PN, Mittelbronn M, Wu T, Seifert V, Senft C (2019) Surgery for glioblastoma in light of molecular markers: impact of resection and MGMT promoter methylation in newly diagnosed IDH-1 Wild-Type glioblastomas. Neurosurgery 84:190–197. 10.1093/neuros/nyy049 29617848 10.1093/neuros/nyy049 PMC6500906

[37] Li YM, Suki D, Hess K, Sawaya R (2016) The influence of maximum safe resection of glioblastoma on survival in 1229 patients: can we do better than gross-total resection? J Neurosurg 124:977–988. 10.3171/2015.5.JNS142087 26495941 10.3171/2015.5.JNS142087

[38] El-Abtah ME, Talati P, Fu M, Chun B, Clark P, Peters A, Ranasinghe A, He J, Rapalino O, Batchelor TT, Gilberto Gonzalez R, Curry WT, Dietrich J, Gerstner ER, Ratai EM (2022) Magnetic resonance spectroscopy outperforms perfusion in distinguishing between pseudoprogression and disease progression in patients with glioblastoma. Neurooncol Adv 4:vdac128. 10.1093/noajnl/vdac128 36071927 10.1093/noajnl/vdac128 PMC9446677

